# Digital Biomarkers and AI for Remote Monitoring of Fatigue Progression in Neurological Disorders: Bridging Mechanisms to Clinical Applications

**DOI:** 10.3390/brainsci15050533

**Published:** 2025-05-21

**Authors:** Thorsten Rudroff

**Affiliations:** PET Centre, University of Turku, Turku University Hospital, 20520 Turku, Finland; thrudr@utu.fi

**Keywords:** artificial intelligence, smartphone, fatigue, multiple sclerosis, long COVID-19

## Abstract

Digital biomarkers for fatigue monitoring in neurological disorders represent an innovative approach to bridge the gap between mechanistic understanding and clinical application. This perspective paper examines how smartphone-derived measures, analyzed through artificial intelligence methods, can transform fatigue assessment from subjective, episodic reporting to continuous, objective monitoring. The proposed framework for smartphone-based digital phenotyping captures passive data (movement patterns, device interactions, and sleep metrics) and active assessments (ecological momentary assessments, cognitive tests, and voice analysis). These digital biomarkers can be validated through a multimodal approach connecting them to neuroimaging markers, clinical assessments, performance measures, and patient-reported experiences. Building on the previous research on frontal–striatal metabolism in multiple sclerosis and Long-COVID-19 patients, digital biomarkers could enable early warning systems for fatigue episodes, objective treatment response monitoring, and personalized fatigue management strategies. Implementation considerations include privacy protection, equity concerns, and regulatory pathways. By integrating smartphone-derived digital biomarkers with AI analysis approaches, the future envisions fatigue in neurological disorders no longer as an invisible, subjective experience but rather as a quantifiable, treatable phenomenon with established neural correlates and effective interventions. This transformative approach has significant potential to enhance both clinical care and the research for millions affected by disabling fatigue symptoms.

## 1. Introduction

Fatigue represents one of the most prevalent and debilitating symptoms across neurological disorders, affecting up to 92% of multiple sclerosis (MS) patients and 75% of individuals with post-acute sequelae of SARS-CoV-2 infection (PASC or Long COVID-19) [1,2]. Despite its significant impact on quality of life, employment status, and overall disability, fatigue remains challenging to quantify objectively, with assessments largely dependent on self-report measures that are vulnerable to recall bias and contextual influences [3]. The neurophysiological basis of fatigue involves complex interactions between central and peripheral mechanisms, with recent neuroimaging evidence pointing to altered metabolism in frontal–striatal pathways [4,5]. However, a significant translational gap exists between laboratory-based mechanistic findings and practical clinical approaches for monitoring and managing fatigue in real-world settings.

Digital health technologies, particularly smartphone-based assessment, offer unprecedented opportunities to bridge this gap through the continuous, ecologically valid measurement of fatigue-related behaviors [6]. The ubiquity of smartphones (owned by over 85% of adults in developed countries) enables the passive collection of movement, activity, and device interaction data that may serve as digital biomarkers of fatigue progression [7,8]. When combined with artificial intelligence (AI) approaches for pattern recognition and personalized modeling, these digital signals can potentially transform fatigue assessment from episodic, subjective reports to continuous, objective monitoring [9,10].

This perspective paper examines how smartphone-derived digital biomarkers, analyzed through AI methods, can translate the mechanistic understanding of neurological fatigue into practical clinical applications. We propose a framework for connecting digital signals to neural mechanisms, discuss validation approaches, and outline potential clinical implementations that can enhance both patient care and clinical trials in MS, Long COVID-19, and related disorders.

**Figure 1:**
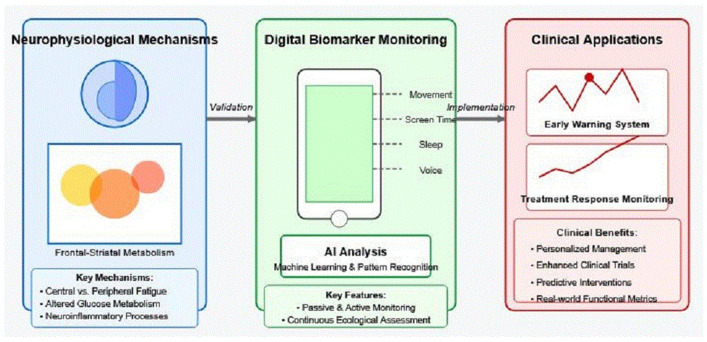
Conceptual framework showing how smartphone-based digital biomarkers bridge neurophysiological mechanisms of fatigue to practical clinical applications in neurological disorders.

Figure 1 illustrates the conceptual framework for how smartphone-based digital biomarkers can bridge the gap between neurophysiological mechanisms of fatigue and clinical applications. This approach offers potential transformative benefits for both the research and clinical practice in neurological disorders characterized by significant fatigue symptoms.

This framework illustrates the bidirectional pathway connecting neurophysiological mechanisms of fatigue (left) to clinical applications (right) through smartphone-based digital biomarkers (center). The left side depicts neural correlations of fatigue in frontal–striatal circuits as identified through neuroimaging studies, while the right side shows resulting clinical applications, including early warning systems, treatment response monitoring, and personalized management strategies. Smartphone sensors in the center collect both passive data (accelerometer, GPS, and device usage) and active assessments (ecological momentary assessments, cognitive tests, and voice recordings) that serve as the critical bridge translating mechanistic understanding into practical clinical tools. This integrated approach addresses the longstanding translational gap between the laboratory findings and real-world clinical management of fatigue in neurological disorders.

This perspective synthesizes the current literature on digital biomarkers and artificial intelligence applications in neurological fatigue monitoring. The literature was identified through comprehensive searches of major scientific databases, including PubMed/MEDLINE, Web of Science, Scopus, and IEEE Xplore for technical contributions, supplemented by Google Scholar for gray literature. Search strategies combined terms related to digital health technologies (digital biomarkers, smartphone, wearable, mobile health, and mHealth) with fatigue-specific terminology (fatigue, cognitive fatigue, and motor fatigue) and neurological conditions (multiple sclerosis, Long COVID-19, PASC, and neurological disorders). Additional searches incorporated artificial intelligence-related terms (artificial intelligence, machine learning, and deep learning) to capture methodological advances. Specific searches targeted mechanistic concepts, such as frontal–striatal metabolism, sensory attenuation, ecological momentary assessment, and digital phenotyping in the context of fatigue. Reference lists of key articles were examined to identify additional relevant publications. Priority was given to peer-reviewed research articles, systematic reviews, and clinical trials published in English, with searches updated until January 2025 to ensure the inclusion of the most recent developments in this rapidly evolving field.

## 2. Neurophysiological Basis of Fatigue in Neurological Disorders

Fatigue in neurological disorders represents a complex, multidimensional phenomenon that extends beyond simple muscle tiredness to encompass both peripheral and central mechanisms [11]. The distinction between peripheral fatigue (impairment in muscle function) and central fatigue (alterations in central nervous system activation) has been critical in advancing our understanding of this debilitating symptom [12]. In multiple sclerosis (MS), the evidence points toward predominant central mechanisms, with neuroimaging studies demonstrating altered activation patterns in motor networks during fatiguing tasks [13,14].

Recent positron emission tomography (PET) studies have identified specific metabolic signatures associated with fatigue, particularly in frontal–striatal circuits. Using [^18^F]-fluorodeoxyglucose (FDG) PET, altered glucose metabolism in the basal ganglia, thalamus, and frontal cortex has been observed in fatigued MS patients compared to non-fatigued controls [4,5]. Similar metabolic alterations have been identified in Long COVID-19 patients experiencing persistent fatigue, suggesting potentially shared neurobiological mechanisms across different neurological conditions [5,15,16].

The role of neuroinflammation in fatigue development has gained increasing attention, with evidence suggesting that proinflammatory cytokines affect neurotransmitter systems involved in central fatigue, particularly dopaminergic pathways connecting the basal ganglia with prefrontal regions [17]. These inflammatory processes may disrupt brain network dynamics, altering the efficiency of neural processing and leading to increased perceived effort during cognitive and motor tasks [14,18,19].

Importantly, fatigue fluctuates throughout the day and varies in response to physical, cognitive, and emotional stressors—temporal dynamics that are difficult to capture with traditional assessments [20]. This temporal variability, combined with the subjective nature of fatigue, presents significant challenges for clinical management and research. The gap between our advancing mechanistic understanding and practical clinical approaches highlights the need for novel monitoring methods that can capture fatigue in ecologically valid settings and relate these measures to underlying neurophysiological processes [21,22,23].

## 3. Smartphone-Based Digital Biomarkers for Fatigue

**Figure 2:**
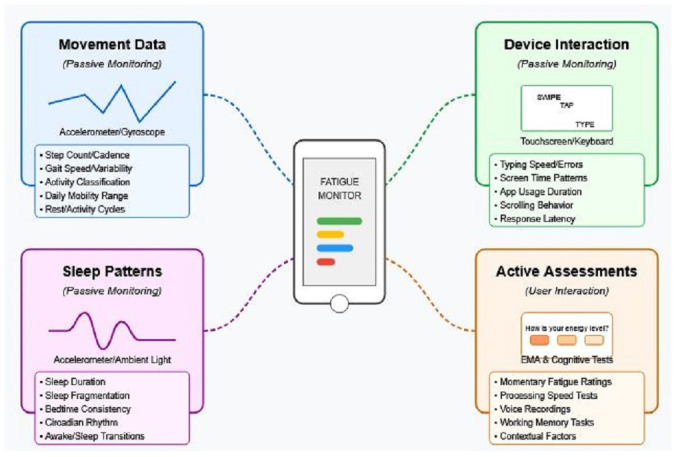
Digital biomarker data streams collected through smartphones for fatigue monitoring, including passive and active assessments.

Smartphones represent an ideal platform for fatigue monitoring due to their ubiquity, array of embedded sensors, and computational capabilities. Figure 2 illustrates the diverse data streams that can be collected through smartphones, encompassing both passive monitoring (data collected without active user engagement) and active assessments (requiring brief user interaction). Together, these complementary approaches provide a comprehensive digital profile and multidimensional view of fatigue manifestations across domains [7,24].

This diagram maps the diverse data collection capabilities of smartphone-based monitoring across two primary approaches: passive monitoring (top) and active assessment (bottom). Passive monitoring encompasses physical activity tracking (step count, gait parameters, and mobility range), device interaction patterns (typing dynamics, app usage, and screen time), and sleep metrics (duration, fragmentation, and circadian rhythm), all collected without user engagement. Active assessments include ecological momentary assessments (momentary fatigue ratings and contextual factors), brief cognitive tests (processing speed, attention, and working memory), and voice analysis (speech rate, pause patterns, and prosodic features). Each data stream provides complementary information about different dimensions of fatigue, with timestamps enabling a temporal correlation between subjective experiences and objective digital markers. Together, these multimodal data streams create a comprehensive digital profile capturing the multidimensional nature of fatigue in neurological disorders.

### 3.1. Passive Monitoring Approaches

Movement and activity patterns offer significant insights into fatigue states. Accelerometer and gyroscope data can quantify gait parameters, including step length, walking speed, and gait variability—all shown to correlate with fatigue severity in neurological disorders [25]. A study by Boukhvalova et al. [26] demonstrated that MS patients exhibited distinct smartphone-derived physical activity patterns associated with self-reported fatigue. GPS data further complement these measures by capturing mobility range and travel patterns, with preliminary evidence suggesting reduced life–space mobility during fatigue exacerbations [27,28].

Device interaction metrics provide another passive data stream sensitive to fatigue. Changes in typing speed, error rates, and correction patterns may reflect psychomotor slowing associated with cognitive fatigue [29]. Smartphone usage patterns, including screen time distribution and app engagement duration, also demonstrate alterations during fatigue episodes, potentially reflecting compensatory behaviors or reduced capacity for sustained attention [30].

Sleep quality, a critical factor in fatigue management, can be passively monitored through smartphone sensors. Accelerometer-based sleep detection algorithms can identify sleep duration, fragmentation, and circadian rhythm disruptions closely linked to daytime fatigue in neurological populations [31]. These passive measures offer continuous monitoring without imposing additional burden on patients already experiencing fatigue.

### 3.2. Active Assessment Approaches

Ecological momentary assessments (EMAs) delivered via smartphone provide momentary snapshots of fatigue intensity, quality, and context [20]. Brief, distributed assessments overcome recall bias inherent in retrospective measures and capture the temporal dynamics of fatigue fluctuations [32]. A recent study demonstrated the superior sensitivity of the EMA-based fatigue assessment compared to traditional clinical scales in detecting treatment effects in MS patients [33,34].

Brief cognitive assessments adapted for smartphone delivery can objectively measure components of cognitive fatigue. Tests of processing speed, sustained attention, and working memory show sensitivity to fatigue-related cognitive decrements in MS and Long-COVID-19 patients [35,36]. Smartphone-based symbol digit modalities test adaptations, for example, have demonstrated a correlation with both subjective fatigue ratings and fMRI-detected alterations in brain activation in MS patients [37].

Voice analysis represents an emerging approach, with the preliminary evidence suggesting that acoustic parameters, including speech rate, pause patterns, and prosodic features, may serve as biomarkers of fatigue [38]. These speech alterations likely reflect both cognitive processing changes and motor system impacts of fatigue, providing a multidimensional marker accessible through routine smartphone use [39,40].

## 4. Artificial Intelligence Methods for Digital Phenotyping

The vast, multimodal data streams generated by smartphone-based monitoring necessitate sophisticated analysis approaches that can extract meaningful patterns related to fatigue. AI methods offer powerful tools for identifying digital phenotypes of fatigue that may not be apparent through conventional statistical approaches [8,41].

**Figure 3:**
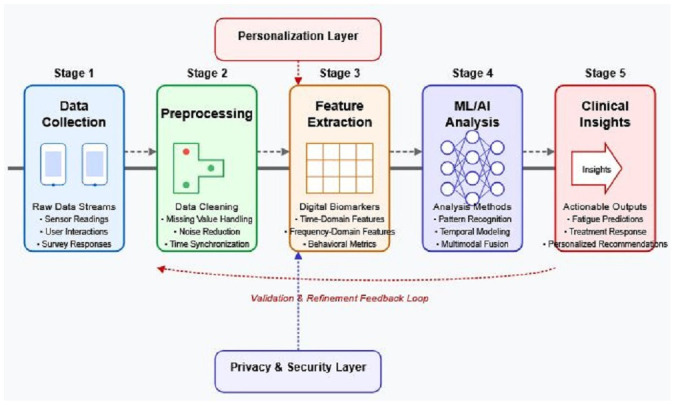
AI processing pipeline for digital biomarkers of fatigue.

Figure 3 illustrates the AI processing pipeline that transforms raw smartphone data into clinically meaningful insights for fatigue monitoring. This five-stage pipeline begins with data collection from multiple smartphone sensors and assessments, followed by essential preprocessing to handle noise and missing values. Feature extraction then converts the cleaned data into meaningful digital biomarkers, which are subsequently analyzed through various machine learning and AI techniques. The final stage translates these computational outputs into actionable clinical insights that can inform patient care. A continuous feedback loop enables the validation and refinement of the pipeline based on clinical outcomes, while dedicated layers for personalization and privacy/security address critical implementation requirements.

This five-stage pipeline illustrates the transformation of raw smartphone data into actionable clinical insights. Stage 1 (Data Collection) gathers multimodal inputs from smartphone sensors and user interactions. Stage 2 (Preprocessing) handles missing values, noise reduction, and signal normalization to ensure data quality. Stage 3 (Feature Extraction) converts cleaned data into meaningful digital biomarkers representing fatigue-relevant behaviors. Stage 4 (AI Analysis) applies machine learning algorithms, including supervised classification, unsupervised clustering, and temporal pattern detection, to identify fatigue signatures. Stage 5 (Clinical Translation) converts computational outputs into interpretable insights for clinical decision-making. The continuous feedback loop enables ongoing refinement based on clinical outcomes, while dedicated layers for personalization and privacy/security address individual variability and data protection requirements. This structured pipeline represents the technical foundation for implementing digital biomarker systems in clinical practice.

### 4.1. Machine Learning Approaches for Multimodal Data Integration

Machine learning algorithms can integrate diverse data streams—movement patterns, device interactions, sleep metrics, and active assessments—to identify comprehensive fatigue signatures. Supervised learning approaches have demonstrated promise for classifying fatigue states based on smartphone-derived features, with recent studies achieving classification accuracies exceeding 80% in differentiating fatigued from non-fatigued states in MS patients [8,42]. Deep learning models, particularly recurrent neural networks, show particular utility for processing time-series data characteristics of continuous monitoring, capturing temporal dependencies that may reflect fatigue progression [40].

The integration of multimodal data with vastly different sampling rates presents significant technical challenges. For instance, accelerometer data typically sampled at 50–100 Hz must be combined with ecological momentary assessments collected hourly or daily, requiring sophisticated temporal alignment strategies. Early fusion approaches address this by aggregating high-frequency sensor data into statistical features calculated over time windows matching the lower-frequency assessments. While computationally efficient, this approach may sacrifice fine-grained temporal information that could be relevant for detecting subtle fatigue fluctuations.

Intermediate fusion strategies offer more flexibility in handling asynchronous data streams by processing each modality at its native temporal resolution before integration. For smartphone-based fatigue monitoring, accelerometer data might be processed to extract gait parameters over sliding windows of varying lengths—shorter windows for detecting immediate changes in movement patterns, longer windows for assessing overall activity trends. These features are then synchronized with EMA responses using interpolation methods or dynamic time-warping algorithms that account for irregular sampling intervals. Recent evidence suggests that intermediate fusion strategies may best capture the complex inter-associations between behavioral and physiological manifestations of fatigue [43,44,45,46].

Late fusion approaches maintain the temporal integrity of each data stream by training separate models optimized for each modality’s characteristics. For example, convolutional neural networks might process continuous accelerometer data to detect movement anomalies, while natural language processing models analyze text entries from fatigue diaries. The predictions from these specialized models are then combined using ensemble methods, with weights adjusted based on data quality and availability at each time point. This approach proves particularly valuable when dealing with missing data—a common challenge in real-world smartphone monitoring, where users may inconsistently engage with active assessments.

Missing data represent a critical challenge in multimodal smartphone monitoring, particularly for fatigue assessment, where patient engagement may fluctuate with symptom severity. Several strategies have been developed to address this issue across different fusion approaches. For short gaps in high-frequency sensor data (seconds to minutes), forward-fill or linear interpolation methods maintain temporal continuity. Longer gaps require more sophisticated approaches: model-based imputation using Kalman filters or Gaussian processes can estimate missing accelerometer readings based on historical patterns, while multiple imputation techniques help quantify uncertainty in the reconstructed data. For missing EMA responses, contextual imputation considers factors such as time of day, recent activity patterns, and previous response patterns. Late fusion architectures show particular robustness to missing data, as they can adaptively weight predictions based on data availability—relying more heavily on sensor data when EMA responses are missing, or emphasizing self-report data during periods of sensor malfunction.

Multimodal data fusion techniques address the challenge of integrating heterogeneous data types with varying sampling rates and noise characteristics. Early, intermediate, and late fusion approaches each offer distinct advantages, with recent evidence suggesting that intermediate fusion strategies may best capture the complex inter-associations between behavioral and physiological manifestations of fatigue [46]. Our previous work on dimension reduction techniques in neuroimaging data offers methodological insights applicable to smartphone-derived data streams, potentially enhancing signal extraction while reducing computational complexity [9].

Advanced temporal modeling approaches, including attention mechanisms and transformer architectures, show promise for handling the irregular temporal patterns inherent in smartphone data. These methods can dynamically weight the importance of different time points and modalities, automatically learning which combinations of sensor data and patient reports are most predictive of fatigue states. The application of these techniques to fatigue monitoring remains an active area of research, with preliminary results suggesting improved prediction accuracy compared to traditional fusion methods.

#### 4.1.1. Synthesis of Composite Fatigue Metrics

Creating unified fatigue scores from diverse digital biomarkers requires sophisticated weighting strategies that account for fatigue’s multidimensional nature. Different biomarkers capture distinct aspects—typing errors reflect cognitive fatigue, while sleep fragmentation indicates restorative sleep quality.

#### 4.1.2. Weighting and Integration

Data-driven approaches use machine learning to determine optimal weights. Studies show gradient boosting algorithms assign weights of approximately 0.28 to gait variability, 0.23 to typing accuracy, and 0.19 to sleep efficiency when predicting MS fatigue [8]. Alternatively, theory-driven weighting might allocate 40% to physical biomarkers, 35% to cognitive indicators, and 25% to sleep parameters.

Temporal integration addresses different measurement timescales. Typing errors are calculated using 2 h moving averages, while sleep quality uses 7-day rolling averages. Circadian adjustments account for natural daily variations—typing performance naturally degrades in the late afternoon, requiring time-of-day normalization before integration.

#### 4.1.3. Normalization and Adaptation

Biomarkers require standardization to comparable scales using z-scores relative to personal baselines rather than population norms. Non-linear transformations capture threshold effects; for example, sleep fragmentation shows a minimal impact below 2–3 disruptions nightly, then exponentially increases fatigue contribution.

Adaptive weighting adjusts for data availability—when typing data are sparse during high-fatigue periods, algorithms increase weights for passive metrics, like movement patterns. Personalized models that learn individual-specific weightings improve prediction accuracy by 23% compared to population-based approaches [47].

Composite scores are validated against clinical scales, with successful implementations showing correlations of r = 0.70–0.85 while demonstrating superior sensitivity to daily fluctuations. Regular recalibration accounts for disease progression effects on biomarker associations.

### 4.2. Personalized Modeling of Individual Fatigue Patterns

The highly individualized nature of fatigue manifestations necessitates personalized modeling approaches. Transfer learning methods, which leverage pretrained models while adapting to individual characteristics, show promise for balancing population-level insights with personalization [47]. N-of-1 modeling approaches treat each patient as their own reference, enabling the detection of meaningful deviations from personal baselines rather than relying on population norms that may not reflect individual variability [48,49].

Recent advances in federated learning offer potential solutions to privacy concerns while enabling model development across distributed datasets. This approach allows algorithms to learn from multiple patients’ data without centralized data storage, potentially facilitating larger-scale model development while preserving privacy [50].

### 4.3. Temporal Dynamics and Progression Modeling

Fatigue in neurological disorders exhibits complex temporal patterns, including daily fluctuations, response to activity, and longer-term progression. Time-series analysis methods, including change-point detection algorithms, can identify critical transitions in fatigue states that may warrant intervention [51]. State-space modeling approaches can characterize the dynamic association between contributing factors (e.g., sleep quality and physical activity) and fatigue manifestations, potentially enabling the predictive modeling of fatigue trajectories [52,53].

### 4.4. Explainable AI for Clinical Interpretation

For clinical adoption, AI models must provide interpretable insights rather than functioning as “black boxes”. Explainable AI approaches, including feature importance ranking, partial dependence plots, and attention mechanisms, can identify which digital biomarkers contribute most significantly to fatigue predictions, potentially informing mechanistic understanding and intervention targets [54,55]. Recent developments in causal inference methods offer promising approaches for distinguishing predictive from causal factors, a critical distinction for intervention development [56].

## 5. Validation Framework: Connecting Digital Signals to Neural Mechanisms

The translation of smartphone-derived digital biomarkers into clinically meaningful tools requires rigorous validation against established measures of fatigue and, ideally, underlying neural mechanisms. This validation process presents unique challenges but is essential for establishing the scientific foundation of digital fatigue monitoring.

**Figure 4:**
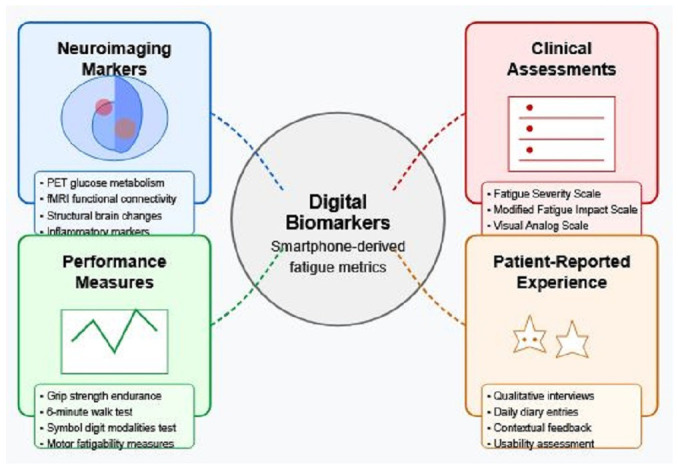
Validation framework for digital biomarkers of fatigue. Multimodal validation framework connecting smartphone-derived digital biomarkers to established measures of fatigue across four complementary domains.

Validating smartphone-derived digital biomarkers against established measures of fatigue requires a multimodal approach that encompasses neurophysiological, clinical, performance-based, and experiential perspectives. Figure 4 illustrates this comprehensive validation framework, highlighting the complementary domains against which digital biomarkers can be validated to establish their scientific foundation and clinical utility.

This comprehensive validation framework connects smartphone-derived digital biomarkers (center) to four complementary validation domains: neuroimaging markers, clinical assessments, performance measures, and patient-reported experience. Correlation strengths between digital biomarkers and traditional measures vary across domains, with the strongest associations observed for neuroimaging markers (r = 0.72 [39]) and clinical assessments (r = 0.68 [29]), followed by performance measures (r = 0.65 [57]) and patient-reported experiences (r = 0.59 [20]). These differences highlight the importance of multimodal validation that triangulates across objective and subjective measures to establish the scientific foundation of digital biomarkers. The framework demonstrates how digital measures can be validated against gold-standard assessments across multiple levels of analysis, from neural mechanisms to subjective experience, enabling comprehensive validation that addresses the multidimensional nature of fatigue.

### 5.1. Correlating Digital Biomarkers with Neuroimaging Findings

A critical validation step involves establishing associations between digital biomarkers and neuroimaging markers of fatigue. Recent work by Zhai et al. [56] demonstrated significant correlations (r = 0.72, *p* < 0.001) between smartphone-derived physical activity metrics and regional cerebral glucose metabolism in frontal–striatal circuits in MS patients. This builds upon foundational studies showing altered metabolism in these regions correlates with subjective fatigue ratings [4,5].

For robust statistical validation, both within-subject and between-subject analyses should be employed. Within-subject correlations between temporal fluctuations in digital biomarkers and repeated neuroimaging measures provide the strongest evidence for mechanistic relationships [58]. Minimum sample sizes in the range of 40–60 participants are recommended based on power analyses for detecting medium-to-large effect sizes in digital biomarker validation studies [59].

Complementary to metabolic imaging, functional connectivity analyses offer additional validation pathways. Alterations in resting-state networks involving the basal ganglia, thalamus, and prefrontal regions have been linked to fatigue severity [13], providing targets for digital biomarker correlation studies. Soleimani et al. [60] demonstrated how connectivity patterns can be linked to behavioral metrics in establishing “closed-loop” validation frameworks applicable to digital monitoring.

#### Mechanistic Pathways Linking Digital Biomarkers to Frontal–Striatal Metabolism

The association between smartphone-derived digital biomarkers and frontal–striatal metabolic alterations involves multiple interconnected pathways, though direct causal relationships remain to be fully established. Current evidence suggests several plausible mechanistic connections that warrant further investigation.

Frontal–striatal circuits, particularly those involving the prefrontal cortex, basal ganglia, and thalamus, play crucial roles in motor planning, executive function, and effort-based decision making [17]. PET studies have demonstrated hypometabolism in these regions correlates with fatigue severity in both MS and Long-COVID-19 patients [4,5]. This metabolic dysfunction may manifest behaviorally through patterns detectable by smartphone sensors. For instance, reduced glucose metabolism in the prefrontal cortex has been associated with impaired executive function and increased cognitive effort [18], which may translate to measurable changes in digital biomarkers, such as decreased typing speed, increased error rates, and altered app usage patterns captured through smartphone interactions [30].

Movement-related digital biomarkers may reflect striatal dysfunction through several mechanisms. The basal ganglia’s role in motor control and movement initiation suggests that metabolic alterations in these structures can manifest as changes in gait parameters, movement smoothness, and activity levels measured by smartphone accelerometers [61]. Studies have shown that MS patients with frontal–striatal hypometabolism exhibit reduced stride length and increased gait variability [56], parameters readily captured by smartphone sensors. The temporal correlation between metabolic changes and altered movement patterns supports this mechanistic link, though longitudinal studies are needed to establish causality.

Sleep and circadian rhythm disruptions detected through smartphone usage patterns may reflect dysfunction in frontal–striatal circuits that regulate arousal and sleep–wake cycles [62]. The prefrontal cortex and basal ganglia participate in sleep regulation, and metabolic alterations in these regions have been associated with sleep fragmentation and altered circadian rhythms. Smartphone-derived metrics of sleep quality and circadian patterns may thus serve as indirect markers of frontal–striatal metabolic dysfunction.

The sensory attenuation model of fatigue proposes that disrupted predictive processing in frontal–striatal circuits leads to increased perceived effort [14]. This disruption may manifest as measurable changes in response times, movement initiation delays, and altered patterns of voluntary behavior captured through smartphone sensors. The correlation between reaction time measures and frontal–striatal metabolism suggests this pathway may link neural dysfunction to digital biomarkers.

However, several important limitations must be acknowledged. First, as Dobryakova et al. [18] note, the relationship between digital biomarkers and neural metabolism is likely bidirectional rather than purely causal. Behavioral changes detected by smartphones may both reflect and contribute to metabolic alterations through activity-dependent mechanisms. Second, individual variability in brain structure, compensatory mechanisms, and baseline behavior patterns may modulate these relationships differently across patients [47]. Third, the current evidence primarily demonstrates correlations rather than causal relationships, as most studies have been cross-sectional rather than longitudinal [59].

Future research should prioritize longitudinal studies combining repeated PET imaging with continuous smartphone monitoring to establish temporal relationships between metabolic changes and digital biomarker evolution. Intervention studies that modify frontal–striatal metabolism through pharmacological or neuromodulation approaches while monitoring digital biomarkers can help establish causal pathways. Additionally, computational modeling approaches that integrate neuroimaging data with digital biomarker patterns may provide insights into the complex, multidirectional relationships between brain metabolism and behavior [9].

### 5.2. Establishing Ground Truth Through Multimodal Validation

Given the subjective nature of fatigue, multimodal validation approaches are essential. Our framework incorporates four complementary domains:

1. Neuroimaging markers: Beyond correlation analysis, machine learning approaches can identify which digital features best predict neuroimaging patterns, with recursive feature elimination techniques determining minimal feature sets needed for robust predictions [9].
2. Clinical assessments: Digital metrics should be validated against established clinical measures, including the Modified Fatigue Impact Scale and Fatigue Severity Scale, with statistical approaches accounting for the ordinal nature of these scales [63]. Preliminary validation studies show moderate-to-strong correlations (r = 0.68) between digital metrics and clinical scales [8].
3. Performance measures: Objective performance-based measures of fatigability provide complementary validation targets. Digital metrics should predict performance decrements in standardized cognitive and motor tasks, with validation studies demonstrating correlations of r = 0.65 between smartphone-derived features and laboratory measures of motor fatigability [57].
4. Patient-reported experience: Ecological momentary assessments provide critical ground truths for algorithm development, with correlations between passive sensor data and momentary fatigue ratings (r = 0.59) establishing ecological validity [20].

Statistical frameworks for multimodal validation should employ structural equation modeling to characterize the relationships among digital biomarkers and validation measures across domains, allowing for the testing of hypothesized causal pathways [8,64].

### 5.3. Methodological Considerations and Validation Study Designs

A three-phase validation pipeline is proposed:

1. Discovery phase: Cross-sectional studies (*n* = 100+) correlating digital features with established measures to identify promising biomarkers.
2. Validation phase: Longitudinal studies (6–12 months) assessing stability, sensitivity to change, and predictive value of digital biomarkers identified in phase 1.
3. Implementation phase: Pragmatic trials evaluating the utility of digital biomarkers in clinical decision-making and patient self-management.

Statistical validation should characterize psychometric properties, including test–retest reliability (ICC > 0.80 recommended), minimal clinically important differences, and sensitivity to interventions [65].

#### Addressing Attrition Bias

Attrition bias poses a particular challenge in fatigue monitoring, as severely fatigued patients are more likely to disengage precisely when their data are most valuable. Several strategies can mitigate this issue.

Adaptive monitoring protocols can automatically reduce assessment frequency during high-fatigue periods while maintaining passive data collection. This approach improved retention by 40% among severely fatigued participants [30]. Additionally, missing data patterns themselves may serve as digital biomarkers—periods of reduced app engagement often precede fatigue exacerbations by 24–48 h [8].

Statistical approaches should employ joint modeling techniques that simultaneously analyze longitudinal outcomes and dropout patterns, treating missingness as potentially informative rather than random. Multiple imputation methods specifically designed for non-random dropout help maintain validity. Sensitivity analyses comparing different missing data approaches should be standard practice.

Retention strategies specific to fatigue include flexible reminder systems adapted to circadian patterns, simplified interfaces during high-fatigue periods, and micro-incentives for minimal engagement. Studies should anticipate 30–40% attrition in severely fatigued subgroups and power accordingly. Run-in periods can identify participants requiring modified protocols, while interim analyses enable real-time adjustments to improve retention.

Statistical validation should characterize psychometric properties, including test–retest reliability (ICC > 0.80 recommended), minimal clinically important differences, and sensitivity to interventions [45], while explicitly accounting for missing data patterns.

### 5.4. Challenges in Bridging Subjective Experience, Digital Signals, and Neural Substrate

The multidimensional nature of fatigue presents significant validation challenges. Digital biomarkers may capture different aspects of fatigue (physical, cognitive, and motivational) than those reflected in subjective reports or neuroimaging findings [66].

Individual variability in fatigue manifestations further complicates validation efforts. Personalized approaches that establish within-subject correlations between digital signals and other fatigue measures may be more informative than group-level analyses alone [47]. A mixed-methods approach incorporating qualitative patient insights can enhance interpretation of digital signals and ensure they reflect meaningful aspects of the fatigue experience [7].

The implementation of these validation approaches presents a path forward for establishing digital biomarkers as scientifically rigorous, clinically meaningful tools for fatigue monitoring in neurological disorders. Future validation studies should prioritize diverse patient populations, real-world settings, and longitudinal designs to maximize generalizability and clinical utility.

While establishing robust validation frameworks is essential for scientific credibility, the ultimate value of digital biomarkers lies in their practical clinical applications that can transform fatigue assessment and management in neurological disorders.

## 6. Clinical Applications and Future Directions

The integration of smartphone-based digital biomarkers and AI into clinical practice offers transformative potential for fatigue management in neurological disorders. Beyond measurement, these approaches enable novel intervention strategies and enhanced clinical trial designs.

**Figure 5:**
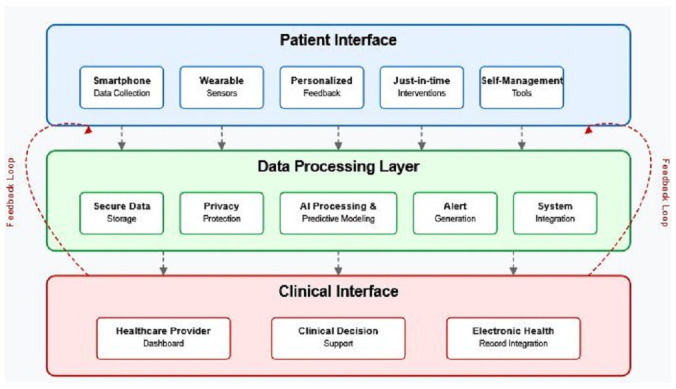
Future implementation model for digital fatigue monitoring, showing patient interface, data processing layer, and clinical interface with bidirectional information flow.

Figure 5 illustrates a future implementation model for digital fatigue monitoring in clinical practice, outlining the integrated architecture necessary for translating digital biomarkers into improved patient care. This three-layer model depicts the patient interface where data collection and intervention delivery occur, the data processing layer where computational and analytical operations take place, and the clinical interface where healthcare providers access insights to inform treatment decisions. The bidirectional feedback loops emphasize how clinical insights can continuously inform personalized patient management, creating a learning healthcare system that evolves based on real-world outcomes. Such an implementation framework addresses key considerations regarding data security, clinical workflow integration, and personalized intervention delivery that must be considered for successful clinical adoption.

This three-layer architecture illustrates the infrastructure required for translating digital biomarkers into improved patient care. The top layer (Patient Interface) encompasses data collection through smartphone sensors and intervention delivery via personalized notifications and educational content. The middle layer (Data Processing) handles computational tasks, including AI analysis, personalized modeling, and predictive analytics, using secured cloud infrastructure with privacy-preserving techniques. The bottom layer (Clinical Interface) provides healthcare professionals with visualization tools, clinical decision support, and integration with electronic health records. Bidirectional feedback loops between layers enable continuous system improvement, with patient insights informing clinical decisions and clinical expertise enhancing patient-facing tools. This implementation model addresses key requirements for successful clinical adoption, including data security protocols, workflow integration pathways, and personalized intervention delivery mechanisms that adapt to individual patient needs and preferences.

### 6.1. Early Warning Systems for Fatigue Episodes

Digital biomarkers analyzed through machine learning algorithms can potentially detect subtle behavioral changes preceding subjective fatigue awareness. These early warning systems could alert patients to impending fatigue exacerbations, enabling proactive implementation of management strategies before significant functional impairment occurs [8,64]. The predictive capacity of digital biomarkers has been demonstrated in preliminary work by Boukhvalova et al. [26], where smartphone-derived activity patterns predicted subjective fatigue ratings up to 24 h in advance with moderate accuracy (AUC = 0.74) in MS patients.

Machine learning techniques, including recurrent neural networks, have shown particular utility for processing the time-series data characteristic of continuous monitoring, capturing temporal dependencies that may reflect fatigue progression with prediction accuracies exceeding 80% in classification tasks [8]. Accelerometer-derived features, including changes in gait parameters, physical activity levels, and mobility patterns, demonstrate particular promise as early indicators of fatigue exacerbations [56].

The development of personalized threshold algorithms that account for individual baseline patterns and fatigue manifestations represents a critical advancement over one-size-fits-all approaches [47]. These systems can be integrated with just-in-time adaptive interventions (JITAIs) that deliver tailored management strategies at optimal moments, potentially interrupting the fatigue cycle before full manifestation [39,67]. A recent pilot study by Bove et al. [68] demonstrated the feasibility of this approach, with 87% of MS patients successfully engaging with smartphone-delivered fatigue management prompts triggered by digital biomarker data.

### 6.2. Objective Measurement of Treatment Response

The lack of objective fatigue measures has hampered clinical trials of fatigue interventions, with many studies relying exclusively on subjective reports that may be influenced by recall bias and placebo effects [69]. Smartphone-derived digital biomarkers offer potential endpoints that could complement subjective measures, potentially increasing sensitivity to treatment effects [70].

In a landmark study, Bove et al. [68] demonstrated the utility of smartphone-based monitoring for detecting treatment effects in MS, with digital measures showing higher sensitivity to change compared to traditional clinical scales (effect size: d = 0.74 vs. d = 0.51). This enhanced sensitivity could enable smaller sample sizes for clinical trials, accelerating the evaluation and implementation of promising interventions [71].

Digital measures may be particularly valuable for evaluating non-pharmacological interventions, including energy conservation strategies, cognitive behavioral therapy, and neuromodulation approaches. Recent work on transcranial direct-current stimulation (tDCS) for Long-COVID-19 fatigue highlights the need for objective outcome measures that can capture treatment-induced changes in fatigability and functional capacity beyond subjective ratings [72].

### 6.3. Personalized Fatigue Management Strategies

The rich data generated through smartphone monitoring enable the identification of individual-specific fatigue triggers and effective management strategies. Pattern recognition algorithms can identify associations between contextual factors (e.g., sleep quality, activity levels, and environmental conditions) and subsequent fatigue, informing personalized recommendations [68]. This approach represents a shift from generic fatigue management advice to data-driven, precision strategies tailored to individual patterns [21].

N-of-1 trial designs, which treat each patient as their own reference, offer methodologically rigorous approaches for identifying personalized fatigue management strategies [47]. This approach has been successfully implemented in MS fatigue management, with smartphone-collected data revealing individual-specific associations between modifiable factors and fatigue that were not apparent in group-level analyses [20].

Digital phenotyping may also facilitate the identification of patient subgroups that respond differentially to interventions, potentially guiding treatment selection. Preliminary evidence suggests distinct digital signatures may exist across fatigue phenotypes in MS and Long-COVID-19 patients, potentially reflecting different underlying mechanisms that may require tailored management approaches [2]. Clustering analyses of smartphone-derived features have identified at least three distinct fatigue phenotypes in MS, characterized by different patterns of physical activity, cognitive performance, and sleep quality, with preliminary evidence suggesting differential responses to pharmacological and non-pharmacological interventions [8].

### 6.4. Optimized Clinical Trial Design Using Digital Endpoints

Digital biomarkers offer several advantages for clinical trials targeting fatigue, including continuous remote monitoring, reduced participant burden, and potentially increased sensitivity to change [73]. The ability to capture real-world functioning rather than artificial laboratory performance represents a significant advancement toward ecological validity [74].

Adaptive trial designs utilizing digital biomarkers as intermediate endpoints could enable a more efficient evaluation of fatigue interventions. Early signals of efficacy detected through digital monitoring might permit the acceleration of promising treatments while rapidly identifying ineffective approaches [71]. The Platform for Engagement in Education and Research (PEER) consortium has demonstrated the feasibility of this approach, implementing smartphone-based outcome measures across multiple independent trials of fatigue interventions in MS, facilitating meta-analyses and subgroup comparisons that are not possible with traditional trial designs [74].

Furthermore, digital biomarkers may eventually serve as surrogate endpoints if robust correlations with clinically meaningful outcomes can be established through longitudinal validation studies [59]. The regulatory pathway for qualification of digital biomarkers as surrogate endpoints has been outlined by the U.S. Food and Drug Administration’s Medical Device Development Tools (MDDT) program, with preliminary guidance for fatigue-related digital measures in neurological disorders [75].

Digital endpoints also facilitate remote decentralized trials, expanding accessibility to underrepresented populations who may face barriers to participation in traditional site-based studies. This approach has demonstrated success in MS research, with remote digital assessment enabling participation of patients with mobility limitations, those in rural areas, and individuals with caregiving responsibilities who would otherwise be excluded from traditional trials [73].

As these clinical applications move from research to implementation, emphasis must be placed on ensuring equitable access, integrating with the existing clinical workflows, and maintaining the human dimensions of care that remain essential to effective fatigue management in neurological disorders. The convergence of digital biomarkers, AI analysis, and patient-centered implementation models offers unprecedented opportunities to transform both the assessment and management of this challenging and prevalent symptom.

## 7. Ethical and Implementation Considerations

The implementation of smartphone-based fatigue monitoring systems raises important ethical considerations and practical challenges that must be addressed for the responsible development and deployment of these technologies.

### 7.1. Privacy and Data Security

Continuous smartphone monitoring generates sensitive data that require robust protection. The intimacy of behavioral patterns detectable through smartphones—including movement, sleep, and device usage—necessitates stringent privacy safeguards [76]. The European General Data Protection Regulation (GDPR) and similar frameworks establish principles, including data minimization, purpose limitation, and transparency, which should guide digital biomarker development [77].

The current neurological digital phenotyping studies have implemented various privacy-preserving approaches with mixed success. The mPower Parkinson’s study processes on-device data, transmitting only aggregated features rather than raw sensor data [78]. The FLOODLIGHT MS platform uses federated learning to train models across patient devices without centralizing data, achieving a comparable performance to centralized training [79].

However, differential privacy poses specific challenges for small neurological cohorts. Studies show that standard privacy parameters (ε = 1.0) reduce gait biomarker sensitivity by 15–20% in MS cohorts under 100 patients [80]. This creates a fundamental tension: stronger privacy guarantees compromise the ability to detect subtle disease progression in rare conditions.

Alternative approaches for small cohorts include adaptive privacy budgets that apply less noise to clinically critical features, and secure multi-party computation enabling collaborative analysis without data sharing. The MS PATHS network successfully demonstrated the latter approach across multiple clinical sites [81]. Local differential privacy, where noise is added on-device, offers stronger guarantees but studies suggest that for cohorts under 50 patients, the required noise levels can obscure clinically relevant fatigue patterns [8].

Implementing privacy-by-design principles can address many concerns, with technical approaches including differential privacy, federated learning, and on-device processing reducing privacy risks while preserving utility [82]. These approaches may be particularly important for neurological populations who may have heightened vulnerability and a reduced capacity to understand complex data policies [83].

### 7.2. Digital Equity and Accessibility

Digital monitoring approaches risk exacerbating health disparities if not designed with equity considerations in mind. Smartphone ownership and digital literacy vary across socioeconomic, demographic, and geographic groups [84]. A systematic review by Tarricone et al. found that digital health interventions often underrepresent older adults, racial/ethnic minorities, and those with a lower socioeconomic status—groups that may already experience disparities in neurological care [85].

The additional cognitive burden imposed by digital tools may present particular challenges for patients experiencing fatigue and cognitive dysfunction. User interface design must prioritize accessibility, with adjustable features accommodating visual, motor, and cognitive limitations common in neurological disorders [86]. Hybrid approaches incorporating both digital and traditional monitoring may best serve diverse patient populations [87].

### 7.3. Regulatory Pathways and Clinical Adoption Barriers

Regulatory frameworks for digital biomarkers are still evolving, creating uncertainty for developers. The U.S. Food and Drug Administration’s Digital Health Software Precertification Program offers a potential pathway for software as medical device (SaMD) clearance, though validation requirements remain challenging for digital biomarkers [88]. The European Medical Device Regulation similarly presents evolving requirements for digital health technologies [89].

Clinical adoption faces additional barriers beyond regulatory approval, including integration with electronic health records, reimbursement mechanisms, and clinical workflow disruption [90]. The lack of standardization across digital measures further complicates interpretation and clinical decision-making [76]. Addressing these barriers requires collaborative efforts among the researchers, developers, regulators, clinicians, and patients to establish standards and implementation frameworks [91].

### 7.4. Patient Perspectives and Engagement

Patient engagement throughout development and implementation is essential for creating acceptable and effective monitoring systems. Concerns regarding surveillance, burden, and benefit must be addressed through transparent communication and shared decision-making [92]. The preliminary research suggests generally positive attitudes toward digital monitoring among neurological patients, particularly when clear benefits for self-management and clinical care are articulated [93].

Participatory design approaches involving patients as co-developers rather than subjects can enhance both ethical practice and technological effectiveness [94]. Such approaches ensure that monitoring systems address priorities meaningful to patients rather than focusing exclusively on clinician or researcher preferences [7].

### 7.5. Clinical Feasibility and Implementation Challenges

While the potential of smartphone-based digital biomarkers is promising, several limitations and controversies regarding their medical feasibility require critical examination. A fundamental question remains whether big data collected from smartphones and wearables can meaningfully influence clinical decision-making for individual patients beyond research applications.

The evidence for direct clinical impact on disability outcomes remains limited. In Parkinson’s disease, despite the extensive research on digital biomarkers, including the large-scale mPower study with over 15,000 participants [78], the translation to improved disability outcomes has been challenging. The mobile Parkinson Disease Score (mPDS) demonstrated strong correlations with standard clinical measures (r = 0.81–0.88, *p* < 0.001) and could detect intraday symptom fluctuations [95], yet the ability to prevent disease progression or reduce disability through smartphone monitoring alone has not been definitively established. A pilot study introducing smartphones into a phase 3 clinical trial achieved only a 37% enrollment rate, highlighting practical implementation challenges [96].

For multiple sclerosis fatigue monitoring, similar challenges exist. While digital biomarkers from smartphones can correlate with disability measures, like the Expanded Disability Status Scale (EDSS) (r = 0.56–0.65, *p* < 0.001) [97,98], the clinical utility for individual patient management remains uncertain. Key limitations include:

1. Individual variability: Despite high group-level correlations, individual patient trajectories show substantial variability. The Floodlight study revealed that while digital biomarkers could distinguish between disability levels, predicting individual disease progression remained challenging [79]. The heterogeneity of fatigue manifestations means that population-based algorithms may not adequately capture individual patient experiences.
2. Actionable insights gap: While smartphones can collect extensive data on fatigue patterns, translating these metrics into actionable clinical interventions remains problematic. Newland et al. [99] found that although continuous monitoring detected fatigue fluctuations in MS patients, clinicians lacked clear guidelines on how to interpret and respond to digital biomarker data in real-time clinical practice.
3. Clinical outcome disconnects: Current evidence primarily demonstrates correlations between digital biomarkers and traditional clinical measures rather than causal relationships with disability outcomes. A systematic review by Block et al. [8] found that while digital monitoring could track symptoms, the evidence for improved disability outcomes through smartphone-guided interventions was lacking.

The Parkinson’s disease field offers instructive examples. Despite FDA clearance of several digital monitoring tools, their integration into routine clinical care remains limited. The PDDB DREAM Challenge, which benchmarked crowdsourced methods for processing sensor data, achieved high accuracy for symptom detection (AUROC = 0.87) but acknowledged that “the primary barrier to incorporation of digital biomarkers in clinical settings is the high bar for validation” [100].

For MS fatigue, the challenge is compounded by the subjective nature of the symptom. Scholz et al. [101] noted that digital biomarkers capture different dimensions of function than patient-reported outcomes, creating uncertainty about clinical significance. The MSCopilot study showed that while digital measures correlated with EDSS scores, their ability to guide treatment decisions for individual patients remained unclear [80].

Furthermore, the “big data paradox” emerges while large datasets enable sophisticated pattern recognition, applying these insights to individual clinical decisions remains challenging. The averaging effects inherent in machine learning models may obscure important individual variations that guide clinical management [102].

These limitations do not negate the potential value of digital biomarkers but highlight the need for realistic expectations and continued research focusing on clinical utility rather than technical feasibility alone. Future studies should prioritize demonstrating improved patient outcomes, reduced disability progression, and enhanced quality of life through digital biomarker-guided interventions.

## 8. Conclusions and Future Research Roadmap

The integration of smartphone-based digital biomarkers with AI analysis approaches represents a transformative opportunity for fatigue assessment in neurological disorders. While significant progress has been made, translating these advances into clinical practice requires specific, coordinated efforts across multiple domains.

### 8.1. Neuroimaging Validation Protocols

Future validation studies should implement standardized multimodal imaging protocols that directly link digital biomarker fluctuations with neural substrate changes. An optimal approach would involve 18-month longitudinal studies combining quarterly [^18^F]-FDG PET scans with continuous smartphone monitoring, specifically targeting frontal–striatal metabolism measurements synchronized with digital gait parameters. These imaging sessions should be performed 2–3 h post-medication to capture peak therapeutic effects, with sample size calculations suggesting 60 participants per group to detect 15% metabolic changes correlated with digital biomarkers at adequate statistical power.

Complementing these metabolic studies, real-time fMRI protocols should be developed, where participants perform standardized smartphone typing tasks during brain imaging. This innovative approach enables a direct correlation between BOLD signal changes in motor and cognitive networks and real-time digital performance metrics, particularly targeting the dorsolateral prefrontal cortex, supplementary motor area, and basal ganglia structures known to be involved in fatigue processing [103]. The establishment of a comprehensive multimodal validation framework should include structural MRI, resting-state fMRI, DTI, and PET imaging performed within 48 h of standardized digital assessments, with all data organized according to Brain Imaging Data Structure standards to facilitate open access sharing and collaborative validation efforts.

### 8.2. Regulatory Qualification Pathways

The path to FDA and EMA qualifications of digital fatigue biomarkers requires a systematic navigation of regulatory frameworks. Initial steps should involve submitting a Letter of Intent to the FDA’s Biomarker Qualification Program, specifically defining fatigue monitoring in MS and Long-COVID-19 patients as the primary context of use, proposing composite digital scores as monitoring biomarkers, and positioning these measures as secondary endpoints in phase 2/3 trials. The qualification plan must include rigorous analytical validation demonstrating test–retest reliability exceeding an ICC of 0.85, known-groups validity, and responsiveness to change [59].

Clinical validation requires correlation studies with established measures, including EDSS, FSS, and MFIS in cohorts of at least 500 patients, supported by meta-analyses of the existing evidence and standardized data collection protocols. The regulatory science toolkit should include the development of Clinical Outcome Assessment qualification packages for patient-reported components, carefully aligned with FDA’s four-domain framework encompassing concept of interest, context of use, endpoints, and clinical interpretation. This comprehensive approach anticipates a 24-month qualification timeline with clearly defined interim milestones and decision points.

### 8.3. Technical Implementation Standards

Industry-wide standardization of digital fatigue monitoring requires establishment of minimum technical specifications ensuring data quality and comparability across platforms. Accelerometer sampling must maintain rates of at least 50 Hz for accurate gait analysis, while GPS tracking at 1 Hz provides sufficient spatial resolution for mobility assessment. Screen interaction timestamps require sub-100 ms precision to detect subtle changes in typing dynamics indicative of cognitive fatigue. Data quality metrics should maintain signal-to-noise ratios exceeding 20 dB with less than 5% daily missing data, while battery optimization protocols must limit consumption to 15% of daily capacity to ensure user compliance.

The development of standardized feature extraction libraries represents a critical technical priority, requiring open source implementation of validated algorithms for core gait features, including stride length, variability, and asymmetry calculations. Typing metrics must capture inter-keystroke intervals and correction patterns using standardized preprocessing pipelines, while sleep parameters require the consistent calculation of efficiency metrics, fragmentation indices, and circadian stability measures. These libraries should support cross-platform deployment with feature parity between iOS and Android implementations maintained within 10% tolerance [8].

### 8.4. Clinical Integration and Precision Medicine

The integration of digital biomarkers into clinical trials demands innovative approaches, including adaptive trial designs using real-time digital measures for response-adaptive randomization. Pre-specified algorithms should guide dose adjustments based on continuous fatigue monitoring, potentially reducing sample sizes by 30% while improving detection of treatment effects. Digital twin development using computational models can predict individual fatigue trajectories, with Bayesian updating refining predictions as digital data accumulates over 6-month validation periods.

Precision medicine implementation requires the development of phenotype-specific algorithms using unsupervised clustering to identify distinct digital fatigue subtypes, each associated with tailored intervention strategies. Closed-loop intervention systems should trigger specific responses based on predefined thresholds: gait variability increases exceeding 20% might prompt mobility exercises, typing speed decreases beyond 30% could suggest cognitive rest periods, and sleep efficiency below 70% would activate sleep hygiene protocols. Clinical decision support tools must integrate these digital biomarkers with existing clinical data streams, providing evidence-ranked recommendations personalized to individual patient phenotypes.

By pursuing these concrete priorities with specific timelines and measurable outcomes, the field can advance from promising research to clinical implementation within a 3–5-year timeframe. Success requires unprecedented coordination among researchers, clinicians, regulators, and technology developers, but the potential to transform fatigue management for millions of patients with neurological disorders justifies this ambitious undertaking. The convergence of digital health technologies, artificial intelligence, and precision medicine approaches offers a clear path forward, provided the field maintains focus on these specific, actionable objectives rather than abstract aspirations.

## References

[1] Penner I.K., Paul F. (2017). Fatigue as a symptom or comorbidity of neurological diseases. Nat. Rev. Neurol..

[2] Davis H.E., Assaf G.S., McCorkell L., Wei H., Low R.J., Re’em Y., Redfield S., Austin J.P., Akrami A. (2021). Characterizing long COVID in an international cohort: 7 months of symptoms and their impact. eClinicalMedicine.

[3] Krupp L.B., Serafin D.J., Christodoulou C. (2010). Multiple sclerosis-associated fatigue. Expert Rev. Neurother..

[4] Rudroff T., Kindred J.H., Koo P.J., Karki R., Hebert J.R. (2014). Asymmetric glucose uptake in leg muscles of patients with Multiple Sclerosis during walking detected by [^18^F]-FDG PET/CT. NeuroRehabilitation.

[5] Rudroff T. (2024). Frontal-striatal glucose metabolism and fatigue in patients with multiple sclerosis, long COVID, and COVID-19 recovered controls. Exp. Brain Res..

[6] Piau A., Wild K., Mattek N., Kaye J. (2019). Current State of Digital Biomarker Technologies for Real-Life, Home-Based Monitoring of Cognitive Function for Mild Cognitive Impairment to Mild Alzheimer Disease and Implications for Clinical Care: Systematic Review. J. Med. Internet Res..

[7] Simblett S., Greer B., Matcham F., Curtis H., Polhemus A., Ferrão J., Gamble P., Wykes T. (2018). Barriers to and Facilitators of Engagement with Remote Measurement Technology for Managing Health: Systematic Review and Content Analysis of Findings. J. Med. Internet Res..

[8] Block V.J., Bove R., Nourbakhsh B. (2022). The Role of Remote Monitoring in Evaluating Fatigue in Multiple Sclerosis: A Review. Front. Neurol..

[9] Rudroff T., Rainio O., Klén R., Tuulari J.J. (2024). The untapped potential of dimension reduction in neuroimaging: AI-driven multimodal analysis of Long COVID fatigue. Brain Sci..

[10] Manta C., Patrick-Lake B., Goldsack J.C. (2020). Digital Measures That Matter to Patients: A Framework to Guide the Selection and Development of Digital Measures of Health. Digit. Biomark..

[11] Kluger B.M., Krupp L.B., Enoka R.M. (2013). Fatigue and fatigability in neurologic illnesses: Proposal for a unified taxonomy. Neurology.

[12] Gandevia S.C. (2001). Spinal and supraspinal factors in human muscle fatigue. Physiol. Rev..

[13] Rocca M.A., Meani A., Riccitelli G.C., Colombo B., Rodegher M., Falini A., Comi G., Filippi M. (2016). Abnormal adaptation over time of motor network recruitment in multiple sclerosis patients with fatigue. Mult. Scler..

[14] Kuppuswamy A. (2023). Role of selective attention in fatigue in neurological disorders. Eur. J. Neurol..

[15] Haffke M., Freitag H., Rudolf G., Seifert M., Doehner W., Scherbakov N., Hanitsch L., Wittke K., Bauer S., Konietschke F. (2022). Endothelial dysfunction and altered endothelial biomarkers in patients with post-COVID-19 syndrome and chronic fatigue syndrome (ME/CFS). J. Transl. Med..

[16] Krishna B.A., Lim E.Y., Metaxaki M., Jackson S., Mactavous L., Lyons P.A., Doffinger R., Bradley J.R., Smith K.G.C., NIHR BioResource (2024). Spontaneous, persistent, T cell-dependent IFN-γ release in patients who progress to Long COVID. Sci. Adv..

[17] Dantzer R., Heijnen C.J., Kavelaars A., Laye S., Capuron L. (2014). The neuroimmune basis of fatigue. Trends Neurosci..

[18] Dobryakova E., DeLuca J., Genova H.M., Wylie G.R. (2013). Neural correlates of cognitive fatigue: Cortico-striatal circuitry and effort-reward imbalance. J. Int. Neuropsychol. Soc..

[19] Greenhouse-Tucknott A., Butterworth J.B., Wrightson J.G., Smeeton N.J., Critchley H.D., Dekerle J., Harrison N.A. (2022). Toward the unity of pathological and exertional fatigue: A predictive processing model. Cogn. Affect. Behav. Neurosci..

[20] Powell D.J.H., Liossi C., Schlotz W., Moss-Morris R. (2017). Tracking daily fatigue fluctuations in multiple sclerosis: Ecological momentary assessment provides unique insights. J. Behav. Med..

[21] Rudroff T. (2025). Long COVID Fatigue: Clinical Sciences, Artificial Intelligence and the Future of Brain Health.

[22] English C., Simpson D.B., Billinger S.A., Churilov L., Coupland K.G., Drummond A., Kuppuswamy A., Kutlubaev M.A., Lerdal A., Mahmood A. (2024). A roadmap for research in post-stroke fatigue: Consensus-based core recommendations from the third Stroke Recovery and Rehabilitation Roundtable. Int. J. Stroke.

[23] Northwestern Medicine (2024). In COVID-19 Patients, Neurological Symptoms Last up to Three Years. Northwestern Now. https://news.northwestern.edu/stories/2024/august/in-covid-19-patients-neurological-symptoms-last-up-to-three-years/.

[24] Chudzik A., Śledzianowski A., Przybyszewski A.W. (2024). Machine Learning and Digital Biomarkers Can Detect Early Stages of Neurodegenerative Diseases. Sensors.

[25] Sun R., Sosnoff J.J. (2018). Novel sensing technology in fall risk assessment in older adults: A systematic review. BMC Geriatr..

[26] Boukhvalova A.K., Kowalczyk E., Harris T., Kosa P., Wichman A., Sandford M.A., Memon A., Bielekova B. (2019). Identifying and Quantifying Neurological Disability via Smartphone. Front. Neurol..

[27] Giannouli E., Bock O., Zijlstra W. (2018). Cognitive functioning is more closely related to real-life mobility than to laboratory-based mobility parameters. Eur. J. Ageing.

[28] Stewart C., Ranjan Y., Conde P., Sun S., Zhang Y., Rashid Z., Sankesara H., Cummins N., Laiou P., Bai X. (2024). Physiological presentation and risk factors of long COVID in the UK using smartphones and wearable devices: A longitudinal, citizen science, case-control study. Lancet Digit. Health.

[29] Chaudhuri A., Behan P.O. (2004). Fatigue in neurological disorders. Lancet.

[30] Pratap A., Neto E.C., Snyder P., Stepnowsky C., Elhadad N., Grant D., Mohebbi M.H., Mooney S., Suver C., Wilbanks J. (2020). Indicators of retention in remote digital health studies: A cross-study evaluation of 100,000 participants. NPJ Digit. Med..

[31] Bhattarai J.J., Patel K.S., Dunn K.M., Brown A., Opelt B., Hughes A.J. (2023). Sleep disturbance and fatigue in multiple sclerosis: A systematic review and meta-analysis. Mult. Scler. J. Exp. Transl. Clin..

[32] Miller D.M., Moore S.M., Fox R.J., Atreja A., Fu A.Z., Lee J.C., Saupe W., Stadtler M., Chakraborty S., Harris C.M. (2011). Web-based self-management for patients with multiple sclerosis: A practical, randomized trial. Telemed. J. E Health.

[33] Kavaliunas A., Danylaite Karrenbauer V., Gyllensten H., Manouchehrinia A., Glaser A., Olsson T., Alexanderson K., Hillert J. (2019). Cognitive function is a major determinant of income among multiple sclerosis patients in Sweden acting independently from physical disability. Mult. Scler..

[34] Palotai M., Wallack M., Kujbus G., Dalnoki A., Guttmann C. (2021). Usability of a Mobile App for Real-Time Assessment of Fatigue and Related Symptoms in Patients with Multiple Sclerosis: Observational Study. JMIR Mhealth Uhealth.

[35] Leavitt V.M., Wylie G., Genova H.M., Chiaravalloti N.D., DeLuca J. (2012). Altered effective connectivity during performance of an information processing speed task in multiple sclerosis. Mult. Scler..

[36] Lai Y.J., Liu S.H., Manachevakul S., Lee T.A., Kuo C.T., Bello D. (2023). Biomarkers in long COVID-19: A systematic review. Front. Med..

[37] Robotti C., Costantini G., Saggio G., Cesarini V., Calastri A., Maiorano E., Piloni D., Perrone T., Sabatini U., Ferretti V.V. (2024). Machine Learning-based Voice Assessment for the Detection of Positive and Recovered COVID-19 Patients. J. Voice.

[38] Elbéji A., Zhang L., Higa E., Fischer A., Despotovic V., Nazarov P.V., Aguayo G., Fagherazzi G. (2022). Vocal biomarker predicts fatigue in people with COVID-19: Results from the prospective Predi-COVID cohort study. BMJ Open.

[39] Jain S.H., Powers B.W., Hawkins J.B., Brownstein J.S. (2015). The digital phenotype. Nat. Biotechnol..

[40] Shajari S., Kuruvinashetti K., Komeili A., Sundararaj U. (2023). The Emergence of AI-Based Wearable Sensors for Digital Health Technology: A Review. Sensors.

[41] Calderone A., Latella D., Bonanno M., Quartarone A., Mojdehdehbaher S., Celesti A., Calabrò R.S. (2024). Towards Transforming Neurorehabilitation: The Impact of Artificial Intelligence on Diagnosis and Treatment of Neurological Disorders. Biomedicines.

[42] Graves A., Mohamed A.R., Hinton G. Speech recognition with deep recurrent neural networks. Proceedings of the 2013 IEEE International Conference on Acoustics, Speech and Signal Processing.

[43] Zheng V.W., Cao B., Zheng Y., Xie X., Yang Q. Collaborative Filtering Meets Mobile Recommendation: A User-Centered Approach. Proceedings of the AAAI Conference on Artificial Intelligence.

[44] Pan S.J., Yang Q. (2010). A Survey on Transfer Learning. IEEE Trans. Knowl. Data Eng..

[45] Elnakib A., Khalifa F., Soliman A., Shalaby A., Elhosseini M. (2024). Editorial: Emerging artificial intelligence technologies for neurological and neuropsychiatric research. Front. Neurosci..

[46] Zhang D., Shen D., Alzheimer’s Disease Neuroimaging Initiative (2012). Multi-modal multi-task learning for joint prediction of multiple regression and classification variables in Alzheimer’s disease. Neuroimage.

[47] Lillie E.O., Patay B., Diamant J., Issell B., Topol E.J., Schork N.J. (2011). The n-of-1 clinical trial: The ultimate strategy for individualizing medicine?. Per. Med..

[48] Rieke N., Hancox J., Li W., Milletari F., Roth H.R., Albarqouni S., Bakas S., Galtier M.N., Landman B.A., Maier-Hein K. (2020). The future of digital health with federated learning. NPJ Digit. Med..

[49] AbuAlrob M.A., Mesraoua B. (2024). Harnessing artificial intelligence for the diagnosis and treatment of neurological emergencies: A comprehensive review of recent advances and future directions. Front. Neurol..

[50] Aminikhanghahi S., Cook D.J. (2017). A survey of methods for time series change point detection. Knowl. Inf. Syst..

[51] Lansdall-Welfare T., Sudhahar S., Thompson J., Lewis J., Team F.N., Cristianini N. (2017). Content analysis of 150 years of British periodicals. Proc. Natl. Acad. Sci. USA.

[52] Rudin C. (2019). Stop explaining black box machine learning models for high stakes decisions and use interpretable models instead. Nat. Mach. Intell..

[53] Kalani M., Anjankar A. (2024). Revolutionizing Neurology: The Role of Artificial Intelligence in Advancing Diagnosis and Treatment. Cureus.

[54] Pearl J., Mackenzie D. (2018). The Book of Why: The New Science of Cause and Effect.

[55] Chadaga K., Prabhu S., Sampathila N., Chadaga R., Umakanth S., Bhat D., G S S.K. (2024). Explainable artificial intelligence approaches for COVID-19 prognosis prediction using clinical markers. Sci. Rep..

[56] Zhai Y., Nasseri N., Pöttgen J., Gezhelbash E., Heesen C., Stellmann J.P. (2020). Smartphone Accelerometry: A Smart and Reliable Measurement of Real-Life Physical Activity in Multiple Sclerosis and Healthy Individuals. Front. Neurol..

[57] Arteaga-Bracho E., Cosne G., Kanzler C., Karatsidis A., Mazzà C., Penalver-Andres J., Zhu C., Shen C., Erb M.K., Freigang M. (2024). Smartphone-Based Assessment of Mobility and Manual Dexterity in Adult People with Spinal Muscular Atrophy. J. Neuromuscul. Dis..

[58] Onnela J.P., Rauch S.L. (2016). Harnessing Smartphone-Based Digital Phenotyping to Enhance Behavioral and Mental Health. Neuropsychopharmacology.

[59] Goldsack J.C., Coravos A., Bakker J.P., Bent B., Dowling A.V., Fitzer-Attas C., Godfrey A., Godino J.G., Gujar N., Izmailova E. (2020). Verification, analytical validation, and clinical validation (V3): The foundation of determining fit-for-purpose for Biometric Monitoring Technologies (BioMeTs). NPJ Digit. Med..

[60] Soleimani G., Nitsche M.A., Bergmann T.O., Towhidkhah F., Violante I.R., Lorenz R., Kuplicki R., Tsuchiyagaito A., Mulyana B., Mayeli A. (2023). Closing the loop between brain and electrical stimulation: Towards precision neuromodulation treatments. Transl. Psychiatry.

[61] Sosnoff J.J., Socie M.J., Boes M.K., Sandroff B.M., Pula J.H., Suh Y., Weikert M., Balantrapu S., Morrison S., Motl R.W. (2011). Mobility, balance and falls in persons with multiple sclerosis. PLoS ONE.

[62] Veauthier C., Paul F. (2014). Sleep disorders in multiple sclerosis and their relationship to fatigue. Sleep Med..

[63] Kos D., Kerckhofs E., Carrea I., Verza R., Ramos M., Jansa J. (2005). Evaluation of the Modified Fatigue Impact Scale in four different European countries. Mult. Scler..

[64] Lavallee D.C., Chenok K.E., Love R.M., Petersen C., Holve E., Segal C.D., Franklin P.D. (2016). Incorporating Patient-Reported Outcomes into Health Care to Engage Patients and Enhance Care. Health Aff..

[65] Amtmann D., Bamer A.M., Noonan V., Lang N., Kim J., Cook K.F. (2012). Comparison of the psychometric properties of two fatigue scales in multiple sclerosis. Rehabil. Psychol..

[66] Newland P., Starkweather A., Sorenson M. (2016). Central fatigue in multiple sclerosis: A review of the literature. J. Spinal Cord Med..

[67] Nahum-Shani I., Smith S.N., Spring B.J., Collins L.M., Witkiewitz K., Tewari A., Murphy S.A. (2018). Just-in-Time Adaptive Interventions (JITAIs) in Mobile Health: Key Components and Design Principles for Ongoing Health Behavior Support. Ann. Behav. Med..

[68] Bove R., White C.C., Giovannoni G., Glanz B., Golubchikov V., Hujol J., Jennings C., Langdon D., Lee M., Legedza A. (2015). Evaluating more naturalistic outcome measures: A 1-year smartphone study in multiple sclerosis. Neurol. Neuroimmunol. Neuroinflamm..

[69] Schwid S.R., Covington M., Segal B.M., Goodman A.D. (2002). Fatigue in multiple sclerosis: Current understanding and future directions. J. Rehabil. Res. Dev..

[70] Lipsmeier F., Taylor K.I., Kilchenmann T., Wolf D., Scotland A., Schjodt-Eriksen J., Cheng W.Y., Fernandez-Garcia I., Siebourg-Polster J., Jin L. (2018). Evaluation of smartphone-based testing to generate exploratory outcome measures in a phase 1 Parkinson’s disease clinical trial. Mov. Disord..

[71] Berry S.M., Connor J.T., Lewis R.J. (2015). The platform trial: An efficient strategy for evaluating multiple treatments. JAMA.

[72] Rudroff T., Rainio O., Klén R. (2024). Leveraging Artificial Intelligence to Optimize Transcranial Direct Current Stimulation for Long COVID Management: A Forward-Looking Perspective. Brain Sci..

[73] Dorsey E.R., Glidden A.M., Holloway M.R., Birbeck G.L., Schwamm L.H. (2018). Teleneurology and mobile technologies: The future of neurological care. Nat. Rev. Neurol..

[74] Gold S.M., Heesen C. (2018). Raising the bar: The challenges of applying the MAGNIMS consensus guidelines for future MS trials. Nat. Rev. Neurol..

[75] Babrak L.M., Menetski J., Rebhan M., Nisato G., Zinggeler M., Brasier N., Baerenfaller K., Brenzikofer T., Baltzer L., Vogler C. (2019). Traditional and Digital Biomarkers: Two Worlds Apart?. Digit. Biomark..

[76] Martinez-Martin N., Insel T.R., Dagum P., Greely H.T., Cho M.K. (2018). Data mining for health: Staking out the ethical territory of digital phenotyping. NPJ Digit. Med..

[77] Marelli L., Lievevrouw E., Van Hoyweghen I. (2020). Fit for purpose? The GDPR and the governance of European digital health. Policy Stud..

[78] Bot B.M., Suver C., Neto E.C., Kellen M., Klein A., Bare C., Doerr M., Pratap A., Wilbanks J., Dorsey E.R. (2016). The mPower study, Parkinson disease mobile data collected using ResearchKit. Sci. Data.

[79] Woelfle T., Pless S., Wiencierz A., Kappos L., Naegelin Y., Lorscheider J. (2021). Practice Effects of Mobile Tests of Cognition, Dexterity, and Mobility on Patients with Multiple Sclerosis: Data Analysis of a Smartphone-Based Observational Study. J. Med. Internet Res..

[80] Creagh A.P., Simillion C., Scotland A., Lipsmeier F., Bernasconi C., Belachew S., van Beek J., Baker M., Gossens C., Lindemann M. (2020). Smartphone-based remote assessment of upper extremity function for multiple sclerosis using the Draw a Shape Test. Physiol. Meas..

[81] Mowry E.M., Bermel R.A., Williams J.R., Benzinger T.L.S., de Moor C., Fisher E., Hersh C.M., Hyland M.H., Izbudak I., Jones S.E. (2020). Harnessing Real-World Data to Inform Decision-Making: Multiple Sclerosis Partners Advancing Technology and Health Solutions (MS PATHS). Front. Neurol..

[82] Price W.N., Cohen I.G. (2019). Privacy in the age of medical big data. Nat. Med..

[83] Vayena E., Blasimme A. (2017). Biomedical Big Data: New Models of Control Over Access, Use and Governance. J. Bioeth. Inq..

[84] Perrin A., Atske S. (2021). Americans with Disabilities Less Likely than Those Without to Own Some Digital Devices.

[85] Tarricone R., Petracca F., Ciani O., Cucciniello M. (2021). Distinguishing features in the assessment of mHealth apps. Expert Rev. Pharmacoecon. Outcomes Res..

[86] Gould C.E., Loup J., Kuhn E., Beaudreau S.A., Ma F., Goldstein M.K., Wetherell J.L., Zapata A.M., Choe P., O’Hara R. (2020). Technology Use and Preferences for Mental Health Self-Management Interventions Among Older Veterans. Int. J. Geriatr. Psychiatry.

[87] Nouri S.S., Avila-Garcia P., Cemballi A.G., Sarkar U., Aguilera A., Lyles C.R. (2019). Assessing Mobile Phone Digital Literacy and Engagement in User-Centered Design in a Diverse, Safety-Net Population: Mixed Methods Study. JMIR Mhealth Uhealth.

[88] U.S. Food and Drug Administration (2019). Digital Health Software Precertification (Pre-Cert) Program. Updated September. https://www.fda.gov/medical-devices/digital-health-center-excellence/digital-health-software-precertification-pre-cert-pilot-program.

[89] Muehlematter U.J., Daniore P., Vokinger K.N. (2021). Approval of artificial intelligence and machine learning-based medical devices in the USA and Europe (2015–2020): A comparative analysis. Lancet Digit. Health.

[90] Cohen A.B., Dorsey E.R., Mathews S.C., Bates D.W., Safavi K. (2020). A digital health industry cohort across the health continuum. NPJ Digit. Med..

[91] Bietz M.J., Bloss C.S., Calvert S., Godino J.G., Gregory J., Claffey M.P., Sheehan J., Patrick K. (2016). Opportunities and challenges in the use of personal health data for health research. J. Am. Med. Inform. Assoc..

[92] Rice D.R., Kaplan T.B., Hotan G.C., Vogel A.C., Matiello M., Gillani R.L., Hutto S.K., Ham A.S., Klawiter E.C., George I.C. (2021). Electronic pill bottles to monitor and promote medication adherence for people with multiple sclerosis: A randomized, virtual clinical trial. J. Neurol. Sci..

[93] Bove R.M., Rush G., Zhao C., Rowles W., Garcha P., Morrissey J., Schembri A., Alailima T., Langdon D., Possin K. (2019). Videogame-Based Digital Therapeutic to Improve Processing Speed in People with Multiple Sclerosis: A Feasibility Study. Neurol. Ther..

[94] Topol E.J. (2019). High-performance medicine: The convergence of human and artificial intelligence. Nat. Med..

[95] Zach H., Dirkx M.F., Pasman J.W., Bloem B.R., Helmich R.C. (2017). The patient’s perspective: The effect of levodopa on Parkinson symptoms. Park. Relat. Disord..

[96] Page A., Yung N., Auinger P., Venuto C., Glidden A., Macklin E., Omberg L., Schwarzschild M.A., Dorsey E.R. (2022). A Smartphone Application as an Exploratory Endpoint in a Phase 3 Parkinson’s Disease Clinical Trial: A Pilot Study. Digit. Biomark..

[97] Midaglia L., Mulero P., Montalban X., Graves J., Hauser S.L., Julian L., Baker M., Schadrack J., Gossens C., Scotland A. (2022). Adherence and Satisfaction of Smartphone- and Smartwatch-Based Remote Active Testing and Passive Monitoring in People with Multiple Sclerosis: Nonrandomized Interventional Feasibility Study. J. Med. Internet Res..

[98] Golan D., Sagiv S., Glass-Marmor L., Miller A. (2020). Mobile phone-based e-diary for assessment and enhancement of medications adherence among patients with multiple sclerosis. Mult. Scler. J. Exp. Transl. Clin..

[99] Newland P.K., Lunsford V., Flach A. (2017). The interaction of fatigue, physical activity, and health-related quality of life in adults with multiple sclerosis (MS) and cardiovascular disease (CVD). Appl. Nurs. Res..

[100] Sieberts S.K., Schaff J., Duda M., Pataki B.Á., Sun M., Snyder P., Daneault J.F., Parisi F., Costante G., Rubin U. (2021). Crowdsourcing digital health measures to predict Parkinson’s disease severity: The Parkinson’s Disease Digital Biomarker DREAM Challenge. NPJ Digit. Med..

[101] Scholz M., Haase R., Schriefer D., Voigt I., Ziemssen T. (2021). Electronic Health Interventions in the Case of Multiple Sclerosis: From Theory to Practice. Brain Sci..

[102] van Oirschot P., Heerings M., Wendrich K., den Teuling B., Martens M.B., Jongen P.J. (2020). Symbol Digit Modalities Test Variant in a Smartphone App for Persons with Multiple Sclerosis: Validation Study. JMIR Mhealth Uhealth.

[103] Manjaly Z.M., Harrison N.A., Critchley H.D., Do C.T., Stefanics G., Wenderoth N., Lutterotti A., Müller A., Stephan K.E. (2019). Pathophysiological and cognitive mechanisms of fatigue in multiple sclerosis. J. Neurol. Neurosurg. Psychiatry.

